# *Salvia miltiorrhiza* in cancer: Potential role in regulating MicroRNAs and epigenetic enzymes

**DOI:** 10.3389/fphar.2022.1008222

**Published:** 2022-09-12

**Authors:** Meng Lu, Xintian Lan, Xi Wu, Xiaoxue Fang, Yegang Zhang, Haoming Luo, Wenyi Gao, Donglu Wu

**Affiliations:** ^1^ School of Pharmacy, Changchun University of Chinese Medicine, Changchun, China; ^2^ Key Laboratory of Effective Components of Traditional Chinese Medicine, Changchun, China; ^3^ School of Clinical Medical, Changchun University of Chinese Medicine, Changchun, China

**Keywords:** Salvia miltiorrhiza, microRNA, apoptosis, histone modifications, combination medication, cancer

## Abstract

MicroRNAs are small non-coding RNAs that play important roles in gene regulation by influencing the translation and longevity of various target mRNAs and the expression of various target genes as well as by modifying histones and DNA methylation of promoter sites. Consequently, when dysregulated, microRNAs are involved in the development and progression of a variety of diseases, including cancer, by affecting cell growth, proliferation, differentiation, migration, and apoptosis. Preparations from the dried root and rhizome of *Salvia miltiorrhiza Bge* (Lamiaceae), also known as red sage or danshen, are widely used for treating cardiovascular diseases. Accumulating data suggest that certain bioactive constituents of this plant, particularly tanshinones, have broad antitumor effects by interfering with microRNAs and epigenetic enzymes. This paper reviews the evidence for the antineoplastic activities of *S. miltiorrhiza* constituents by causing or promoting cell cycle arrest, apoptosis, autophagy, epithelial-mesenchymal transition, angiogenesis, and epigenetic changes to provide an outlook on their future roles in the treatment of cancer, both alone and in combination with other modalities.

## Introduction

*Salvia miltiorrhiza* is a common herbal beverage in Traditional Chinese Medicine (TCM). *S. milthiorrhiza* belongs to the Lamiaceae family, and its medicinal parts are the roots or rhizomes (Zhang et al., 2022). *S. milthiorrhiza* has been widely used in TCM for the treatment of cardiovascular disease (CVD) (Li et al., 2018; Ren et al., 2019). For instance, Danshen-Honghua is a commonly used herb pair for promoting blood circulation and removing blood stasis, and *S. milthiorrhiza* often appears in some TCM compounds used for cardiovascular and cerebrovascular diseases, such as Danhong Huayu Oral Liquid, Danhong Injection, and Xinning Tablets (Wang et al., 2019b).

**FIGURE 1:**
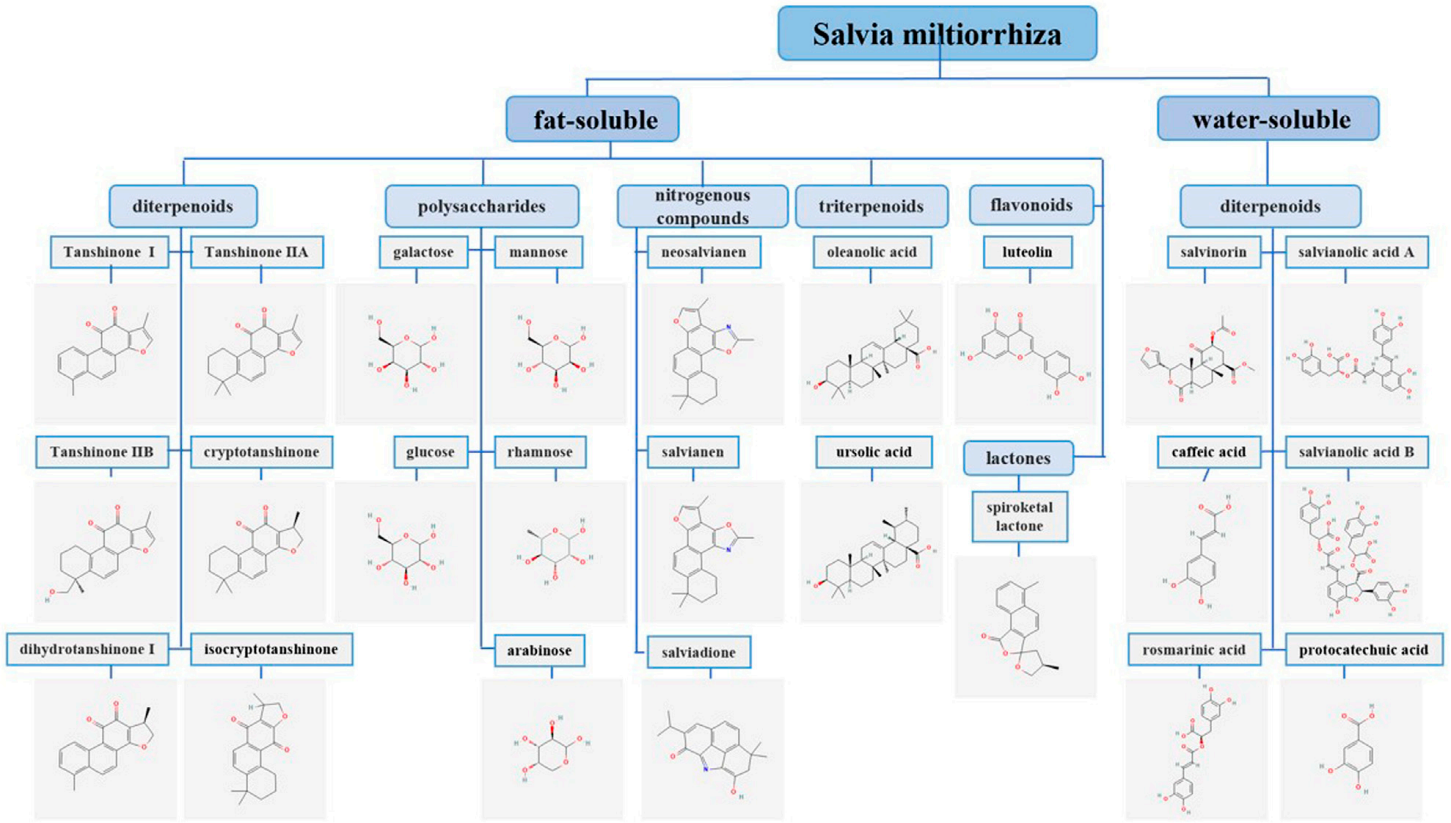
Classification and chemical structures of *S. miltiorrhiza* bioactive compounds. The chemical structures of the *S. miltiorrhiza* constituents included in this publication were obtained from PubChem and drawn with the ChemDraw program.

*S. milthiorrhiza* improves microcirculation, protects the nervous system, and dilates blood vessels, and it also has antitumor properties (Tao et al., 2018). Research has shown that the compounds in *S. milthiorrhiza* are mainly divided into two categories as follows: fat-soluble, which mainly include diterpenoids, triterpenoids, flavonoids, nitrogenous compounds, lactones, and polysaccharides; and water-soluble, which mainly include phenolic acids (Su et al., 2015). Diterpenoids mainly include tanshinones, such as tanshinone I (tan I), tanshinone IIA (tan IIA), tanshinone IIB (tan IIB), cryptotanshinone (CPT), dihydrotanshinone I (DHT I), and isocryptotanshinone (Tian et al., 2021). The water-soluble compounds in *S. milthiorrhiza* are primarily salvianolic acid A (Sal A), salvianolic acid B (Sal B), salvinorin, caffeic acid, rosmarinic acid, and protocatechuic acid (Xin et al., 2020). Most of the triterpenoids are derivatives of oleanolic acid (OA) and ursolic acid (UA) (Tung et al., 2017). The flavonoids in *S. milthiorrhiza* are mainly luteolin (LUT) and its derivatives (Salinas-Arellano et al., 2020). The polysaccharides in *S. milthiorrhiza* are mainly composed of mannose, rhamnose, arabinose, glucose, and galactose (Han et al., 2019). The nitrogen-containing compounds that have been isolated from *S. milthiorrhiza* include neosalvianen, salvianen, and salviadione. In addition, *S. milthiorrhiza* also contains lactone compounds, such as Dan shen spiroketal lactone (Liang et al., 2020; Wang et al., 2020) (Figure 1). Cancer is a disease that seriously endangers human health. According to the latest data released by the World Health Organization’s International Agency for Research on Cancer (IARC), the number of new cancer cases worldwide in 2020 exceeded 19.3 million, and China ranks 68th in the world in cancer incidence rate (Sung et al., 2021). Many studies have reported that the chemical components in *S. milthiorrhiza,* such as tan IIA, have good antitumor activity. In addition to inhibiting the proliferation, blocking the cell cycle, inducing apoptosis, and inducing autophagic death of various types of tumor cells, tan IIA also significantly inhibits cell migration and invasion (Cao et al., 2018; Fang et al., 2020). Tan I and LUT also exert antitumor effects through mediating apoptosis and cell cycle arrest (Anson et al., 2018; Li et al., 2018a).

## Antitumor activities of *S. miltiorrhiza*

### Inhibition of proliferation

*S. miltiorrhiza* bioactive compounds*,* such as tan IIA and CPT, effectively inhibit tumor growth (Chen et al., 2014) and suppress tumor cell proliferation *in vitro* and *in vivo* (Lin et al., 2015; Zhang et al., 2019c). For example, tan IIA inhibits tumor growth in acute promyelocytic leukemia NB4 cell xenograft mice (Zhang et al., 2016c). Liu et al. (2020) reported that CPT inhibits the phosphatidylinositol-3-kinase (PI3K)/protein kinase B (Akt) pathway by inducing the expression of phosphatase and tensin homologue (PTEN), which represses tumor growth in 5637 cell xenograft mice. Furthermore, tanshinol represses U2-OS tumor growth in zebrafish larvae in a dose-dependent manner (Yu et al., 2022). In addition, previous studies have demonstrated that tan IIA suppresses the proliferation of different types of tumor cells in a time- and dose-dependent manner *in vitro*, including gastric cancer (GC; SNU-638, MKN1 and AGS cells) (Zhang et al., 2018) and non-small cell lung cancer (NSCLC; A549 and H292 cells) (Qi et al., 2022). Additionally, it has been reported that tan IIA reduces the phosphorylation of insulin-like growth factor 1 receptor (IGF-1R) and its downstream PI3K/Akt and extracellular signal-regulated kinase 1/2 (ERK1/2) pathways, resulting in the suppression of pheochromocytoma growth (PC12 cells) (Wang et al., 2018a). Other tanshinones also inhibit the development of tumors. For example, tan I suppresses colorectal cancer (CRC) cell proliferation by decreasing aurora A-mediated upregulation of p53 or downregulation of survivin expression to induce apoptosis in HCT116 and SW480 cells (Lu et al., 2016). In addition, CPT enhances the cytotoxicity of CD4^+^ T cells and prevents the development of H446 cells by upregulating the levels of phosphorylated JAK kinase 2 (p-JAK2) and phosphorylated signal transducer and activator of transcription 4 (p-STAT4) (Man et al., 2016). In addition, other components of *S. miltiorrhiza* have been reported to have antitumor properties. For example, LUT suppresses the viability of RPMI-8226 multiple myeloma cells by enhancing the expression of cleaved caspase 3 and microtubule-associated protein light chain 3 (LC3)-II/I (Chen et al., 2018). LUT also inhibits xenograft tumor growth by reducing Ki-67, p-LIMK, and p-cofilin expression (Zhang et al., 2021). Furthermore, salvianolic acid inhibits tumor cell proliferation. Sal A reduces the viability of NSCLC cells (Jin et al., 2021), and Sal B suppresses cell proliferation *via* regulating the Akt/mTOR pathway in DDP-resistant GC cells (Wang et al., 2021a).

The cell cycle is the foundation of cell proliferation, and blockade of the cell cycle prevents tumor development. It has been reported that tan IIA inhibits the proliferation of ovarian cancer (Zhou et al., 2020), cervical cancer (Pan et al., 2010), and GC (Chen et al., 2012) cells by arresting the cell cycle in the G2/M phase. In contrast, tan IIA arrests the cell cycle in lung cancer at the G0/G1 phase *via* upregulating the levels of p53 and p21 as well as downregulating the levels of cyclin D1, cyclin E1, and cyclin-dependent kinase 2 (CDK2) (Lee et al., 2016). Furthermore, tan IIA arrests the cell cycle in prostate cancer in the G0/G1 phase through increasing the expression of p21, p26, and p27 as well as decreasing the expression of cyclin D1, CDK2, and p-Rb (Chiu et al., 2013). Therefore, we propose that the different effects of tan IIA on the cell cycle may be related to cell types. Studies have indicated that other components of *S. miltiorrhiza* also block the cell cycle at different phases in various tumors cells. For example, LUT inhibits cell proliferation of bladder cancer and lung cancer through arresting the cell cycle in the G2/M phase and G1 phase, respectively (Iida et al., 2020; Zhang et al., 2021). LUT blocks the cell cycle in the G2/M phase through upregulating p21^Waf1/Cip1^ and p27^Kip1^ as well as downregulating cyclin A and D1 in bladder cancer T24 cells (Iida et al., 2020). In addition, LUT reduces the expression of cyclin D1 and cyclin D3, thereby inducing cycle arrest of lung cancer cells in the G1 phase (NCI-H1975 and NCI-H1650 cells) (Zhang et al., 2021). Moreover, OA reduces the levels of cyclin D1, CDK1, CDK2, CDK4, and CDK7 but increases the expression of p27, which in turn blocks the cell cycle of HeLa cells in the sub-G1 phase, thereby inhibiting cervical cancer cell growth (Edathara et al., 2021). Moreover, Sal B arrests the cell cycle of A549 cells in the G0/G1 phase by regulating cyclin B1 and p21, thereby repressing A549 cell proliferation (Han et al., 2022). Thus, these findings demonstrate that *S. miltiorrhiza* bioactive compounds suppress the proliferation of various cancers by driving cell cycle arrest, indicating the potential antitumor usage of *S. miltiorrhiza* bioactive compounds in the clinic.

### Promotion of apoptosis

Apoptosis is an autonomous and orderly cell death process controlled by genes that occurs after cells are stimulated by physiological or pathological signals, and tumors have the ability to resist apoptosis *via* multiple strategies (Pistritto et al., 2016; Huang et al., 2021a). The antitumor effect of *S. miltiorrhiza* bioactive compounds has been linked to the induction of apoptosis in multiple studies (Sun et al., 2021). For example, tan IIA induces apoptosis of ovarian cancer A2780 cells by regulating the PI3K/Akt/c-Jun N-terminal kinase (JNK) signaling pathway, which increases the expression of caspase 3, caspase 8, and caspase 9 but decreases the expression of B cell lymphoma 2 (Bcl-2) family proteins (Bcl-w and Bcl-1L) (Zhang et al., 2019a). Tan IIA has also been shown to promote tumor necrosis factor (TNF)-related apoptosis-inducing ligand (TRAIL)-induced apoptosis in glioblastoma by upregulating death receptor 5 (DR5) and blocking STAT3-mediated survivin downregulation (Zhou et al., 2021). Moreover, tan I induces leukemia cell apoptosis *via* inactivation of the PI3K/Akt/survivin signaling pathway, which disrupts the membrane potential, increases Bcl-2-related X (Bax) expression, and activates caspase 3 (Liu et al., 2010). Tanshinone analog 2-((Glycine methyl ester) methyl)-naphtho (TC7) induces cell apoptosis by regulating apoptosis-associated genes, such as p53, ERK1, Bax, p38, Bcl-2, caspase 8, cleaved caspase 8, and poly (ADP-ribose) polymerase 1 (PARP1), as well as the phosphorylation levels of ERK1 and p38 (Wang et al., 2019). Additionally, LUT has been reported to promote apoptosis in a variety of tumor cells, including ovarian teratomas (Liu et al., 2019) and melanomas (Yao et al., 2019), by decreasing Bcl-2 expression, increasing Bax expression, and inhibiting the PI3K/Akt signaling pathway. Moreover, Sal B induces osteosarcoma MG63 cell apoptosis by increasing the expression of cleaved caspase 3, phosphorylated-p38 mitogen-activated protein kinase (p-p38 MAPK), and phosphorylated-p53 (p-p53) (Zeng et al., 2018).

*S. miltiorrhiza* bioactive compounds induce apoptosis of various tumor cells through the mitochondrial pathway (Ye et al., 2017). Tan IIA regulates mitochondrial fission through the JNK/mitochondrial fission factor (MFF) axis, which in turn upregulates mitochondrial pro-apoptotic proteins (Bax and Bad), activates caspase 9, and leads to colon cancer SW837 cell apoptosis (Jieensinue et al., 2018). Reactive oxygen species (ROS) interact with membrane receptors and transcription factors/repressors to activate endogenous and exogenous apoptotic signaling pathways, thereby inducing apoptosis (Su et al., 2019). DHT provokes mitochondrial dysfunction in the early stage by decreasing mitochondrial membrane permeability (MMP) and ROS levels, leading to cell death in colon cancer cells (Wang et al., 2013). Moreover, DHT I stimulates the release of both cytochrome c and apoptosis inducing factor (AIF) in HCT116 cells, which downregulates Bcl-xl and upregulates Bax, thereby inducing apoptosis (Wang et al., 2015). Another *S. miltiorrhiza* component, LUT, promotes apoptosis in canine osteosarcoma cells by generating ROS, increasing loss of MMP, and reducing cytosolic Ca^2+^ concentration (Ryu et al., 2019). In addition, CPT upregulates Bax and cleaved caspase3 but downregulates the anti-apoptotic protein, Bcl-2, which reduces the level of ROS, thereby inducing apoptosis in human melanoma A375 cells (Ye et al., 2016). Furthermore, Sal B induces mitochondria-mediated apoptosis in human colon cancer cells by reducing the mitochondrial potential and increasing mitochondrial cytochrome c release (Gong et al., 2016). Collectively, inducing mitochondria-dependent apoptosis is one of the important ways for *S. miltiorrhiza* bioactive compounds to exert an antitumor effect.

### Inhibition of migration and invasion

Metastasis and invasion are the main manifestations of tumor malignancy, and they are key factors affecting cancer prognosis (Suhail et al., 2019). Tumor cell invasion and metastasis are complicated biological processes that involve the interplay of many growth factors, cytokines, enzyme systems, matrix metalloproteinases, signaling pathways, and epithelial-mesenchymal transition (EMT) (Patriarca et al., 2020). Many studies have reported that numerous *S. miltiorrhiza* bioactive compounds affect tumor metastasis and invasion *via* regulating angiogenesis, regulating tumor cell EMT, and blocking matrix metalloproteinases from hydrolyzing the basement membrane (Zhang et al., 2012). For instance, tan IIA blocks the nuclear factor kappa-B (NF-κB) signaling pathway to inhibit the activity of MMP2 and MMP9, thereby preventing the metastasis and invasion of human hepatoma cells and hepatocellular carcinoma (HCC) cells (Shan et al., 2009; Yuxian et al., 2009; Zhang et al., 2016d).

New blood vessels in tumor tissue provide a source of nutrients for cell proliferation and are also a channel for tumor cell migration (Jiang et al., 2020). Thus, inhibiting tumor angiogenesis has become one of the current antitumor strategies (Fu et al., 2020). Rapid tumor cell proliferation generates hypoxia in local tumor tissue, which activates and upregulates hypoxia-inducible factor 1α (HIF-1α), resulting in the upregulation of the expression of pro-angiogenic factors, such as vascular endothelial growth factor (VEGF), to promote angiogenesis (Keeley et al., 2010). VEGF is a potent angiogenic factor that increases vascular permeability, enabling endothelial cell migration and inducing angiogenesis (Melincovici et al., 2018). Many components of *S. miltiorrhiza* have been demonstrated to suppress tumor cell invasion and metastasis *via* regulating HIF-1α- and VEGF- mediated signaling pathways (Macklin et al., 2017). Tan IIA, tan I, and LUT inhibit the secretion of VEGF under hypoxia by downregulating HIF-1α expression, further inhibiting the growth of new blood vessels and the metastasis and invasion of HCC cells, CRC cells, breast cancer cells, human endothelial progenitor cells, and human umbilical vein endothelial cells (Nizamutdinova et al., 2008; Li et al., 2015; Lee et al., 2017; Sui et al., 2017; Fang et al., 2018; Li et al., 2019; Xue et al., 2019; Zhou et al., 2020c).

During EMT, cells transition from an epithelial state to a mesenchymal state, enabling tumor cells to invade and metastasize (Pastushenko and Blanpain 2019). In the EMT process, epithelial proteins are up- or downregulated, and these proteins are identified as EMT markers. The occurrence of EMT is indicated by decreased E-cadherin and keratin as well increased mesenchymal markers, including vimentin, fibronectin, N-cadherin, and α-SMA (Zeisberg and Neilson 2009). Salvinorin and DHT prevent EMT in colon cancer cells and triple-negative breast cancer (TNBC) cells by increasing E-cadherin levels and decreasing N-cadherin and vimentin levels (Kashyap et al., 2021; Cao et al., 2022).

Numerous studies have shown that *S. miltiorrhiza* bioactive compounds reverse the levels of EMT markers through a complex pathway, including transcription factors, which inhibit the EMT process in tumor cells (Pastushenko and Blanpain 2019). TGF-β is a key factor in inducing EMT in the process of tumor cell proliferation, and elevated levels of TGF-β regulate the expression of EMT markers, which in turn induces EMT (Ali et al., 2015). It has been reported that Sal B and Sal A inhibit the EMT of renal tubular epithelial cells and endothelial cells, respectively, by inhibiting the expression of TGF-β and reversing the expression of EMT markers (Wang et al., 2010; Zhao et al., 2017). YAP/TAZ, a co-activating transcription factor, translocates into the nucleus and binds to transcription factors, such as Twist and Snail, to promote gene transcription (Noguchi et al., 2018; Ma et al., 2019). LUT inhibits the EMT in TNBC cells by inhibiting the transcription activity of YAP/TAZ as well as preventing it from entering the nucleus, further upregulating the protein levels of E-cadherin and downregulating the protein levels of fibronectin, N-cadherin, and vimentin (Cao et al., 2020).

**FIGURE 2:**
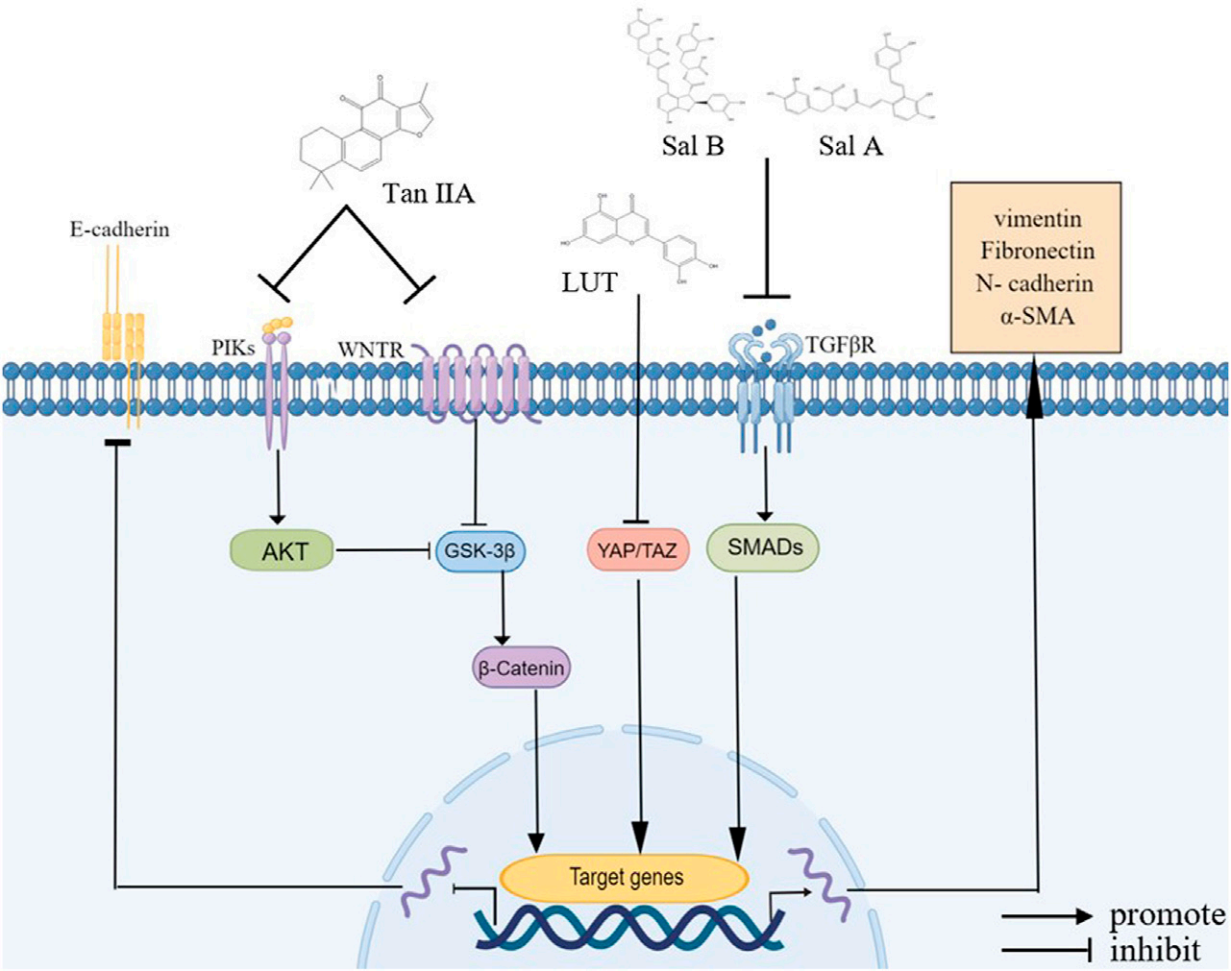
Bioactive compounds of *S. miltiorrhiza* involved in EMT regulation. Tan IIA, LUT, Sal A, and Sal B modulate various signaling pathways, including the NF-κB signaling pathway, VEGF signaling pathway, β-catenin signaling pathway, and PI3K/Akt signaling pathway, and they upregulate E-cadherin and downregulate N-cadherin, α-SMA, vimentin, and fibronectin.

In addition, β-catenin, a cell adhesion regulator, combines with E-cadherin protein on the cell membrane to attach it to the actin cytoskeletal protein, thereby increasing cell adhesion (Shang et al., 2017). GSK-3β is an evolutionarily conserved serine/threonine (S/T) kinase that phosphorylates and degrades cytoplasmic β-catenin, which is regulated by β-arrestin1 and the PI3K/Akt pathway (Yu et al., 2021). Studies have shown that tan IIA increases the expression of GSK-3β by inhibiting the β-arrestin1 and PI3K/Akt signaling pathways, further reducing the nuclear localization of β-catenin and blocking the EMT in CRC and HCC cells (Zhang et al., 2019; Song et al., 2020). Moreover, adhesion factors, such as CCL2, are critical in the EMT process, and the reduction of adhesion factor expression leads to reduced adhesion between transitional cells and adjacent epithelial cells, which causes epithelial cells to lose apical basal cell polarity, thereby promoting the EMT process (Jin 2020; Zou et al., 2020). Studies have shown that tan IIA suppresses the EMT in bladder cancer cells by downregulating CCL2 *via* blocking the STAT3 pathway (Huang et al., 2017) (Figure 2).

In conclusion, *S. miltiorrhiza* bioactive compounds inhibit tumor cell metastasis and invasion through different pathways, including inhibiting the activity of MMP proteins, tumor angiogenesis, and the EMT process.

### Regulation of autophagy

Autophagy is a type of programmed cell death, and it is a target in cancer treatment (Rodrigues et al., 2020). Investigations have revealed that *S. miltiorrhiza* bioactive compounds, such as tan IIA, tan I, CPT, DHT, and LUT, induce autophagy, thereby inhibiting cancer cell proliferation (Fu et al., 2020a).

One of the critical pathways to induce autophagy is the conversion of the soluble form of microtubule-associated protein light chain 3-I (LC3-I) to lipid-soluble LC3-II, which is involved in the formation of autophagosomes, thereby inducing autophagy (Wu et al., 2015). *In vitro* experiments have shown that CPT and DHT induce autophagy by increasing the accumulation of LC3-II in CRC, which induces tumor cell death (Hu et al., 2015).

In addition, the coordinated action of the unc-51-like kinase 1 (ULK1) complex and Beclin 1 functions in driving autophagy (Russell et al., 2013). Beclin 1 is one of the key regulators of autophagy, which participates in the initiation of autophagosome formation, thereby promoting autophagy (Funderburk et al., 2010). For example, LUT induces autophagy in lung cancer cells, HCC cells, and skin squamous cell carcinoma cells by upregulating Beclin 1 expression, subsequently inhibiting tumor cell growth (Verschooten et al., 2012; Park et al., 2013; Potočnjak et al., 2020). Moreover, the ULK1/2 complex is activated in the early stage of autophagy and cooperates with Beclin 1 to initiate autophagosome formation and induce autophagy (Parzych and Klionsky 2014). Furthermore, tan I activates the ULK1 complex and upregulates the expression level of Beclin 1, which subsequently induces autophagy and prevents the growth of breast and liver cancer cells (Zheng et al., 2020).

Mechanistic target of rapamycin (mTOR) is a S/T kinase, which exists in two distinct complexes called mTOR complex 1 (mTORC1) and 2 (mTORC2), and mTORC1 is a key regulator of autophagy (Hua et al., 2019). The activity of mTORC1 reflects the nutritional status of the cell (Kim et al., 2011). Under nutrient-rich conditions, mTORC1 inhibits the autophagy-promoting kinase activity of the ULK1 complex by mediating specific site phosphorylation of ULK1 (Ser637 and Ser757) (Karabiyik et al., 2021). During periods of starvation and cellular stress, mTORC1 activity is inhibited and dissociates from ULK1, resulting in dephosphorylation of ULK1 (Zachari and Ganley 2017). At the same time, the ULK1 complex becomes active through autophosphorylation at Thr180, initiating autophagy (Zachari and Ganley 2017). Studies have shown that tan I inhibits the activity of mTORC1 under starvation conditions, subsequently increasing the activity of the ULK1 complex and promoting the occurrence of autophagy in breast and liver cancer cells (Zheng et al., 2020).

The mTOR pathway is regulated by multiple signaling pathways in autophagy, such as the PI3K/Akt, MAPK/MEK/ERK1/2, and AMPK pathways (Querfurth and Lee 2021). The PI3K/Akt and MAPK/MEK/ERK1/2 signaling pathways have a positive regulatory effect on mTOR, and blocking the above pathways causes autophagy, eventually leading to tumor cell death (Xu et al., 2020a). It has been reported that tan I and tan IIA induce autophagy by inhibiting the PI3K/Akt/mTOR pathway, further inhibiting the proliferation of tumor cells (including ovarian cancer cells, glioma cells, acute promyelocytic leukemia NB4 cells, acute mononuclear leukemia cells, oral squamous cell carcinoma (OSCC) cells, and melanoma cells) (Ding et al., 2017; Li et al., 2017; Qiu et al., 2018; Zhang et al., 2019c; Zhou et al., 2020b; Pan et al., 2021). In addition, tan IIA also drives autophagy through inhibiting the MEK/ERK/mTOR signaling pathway, thereby suppressing the proliferation of colon cancer cells (Qian et al., 2020).

In contrast, the AMPK pathway negatively regulates mTOR. Phosphorylated AMPK inhibits the activity of mTORC1, which indirectly increases the activity of the ULK1 complex, thereby promoting the occurrence of autophagy (Li and Chen 2019). Tan IIA activates AMPK phosphorylation to inhibit mTOR phosphorylation, thereby inducing autophagic death of leukemia cells (Yun et al., 2014). Moreover, under starvation conditions, AMPK activation directly catalyzes the phosphorylation of ULK1 to promote autophagy (Karabiyik et al., 2021). According to a previous study, tan I activates the AMPK signaling pathway to active the ULK1 complex, which subsequently induces autophagy and prevents the growth of breast and liver cancer cells (Zheng et al., 2020).

**FIGURE 3:**
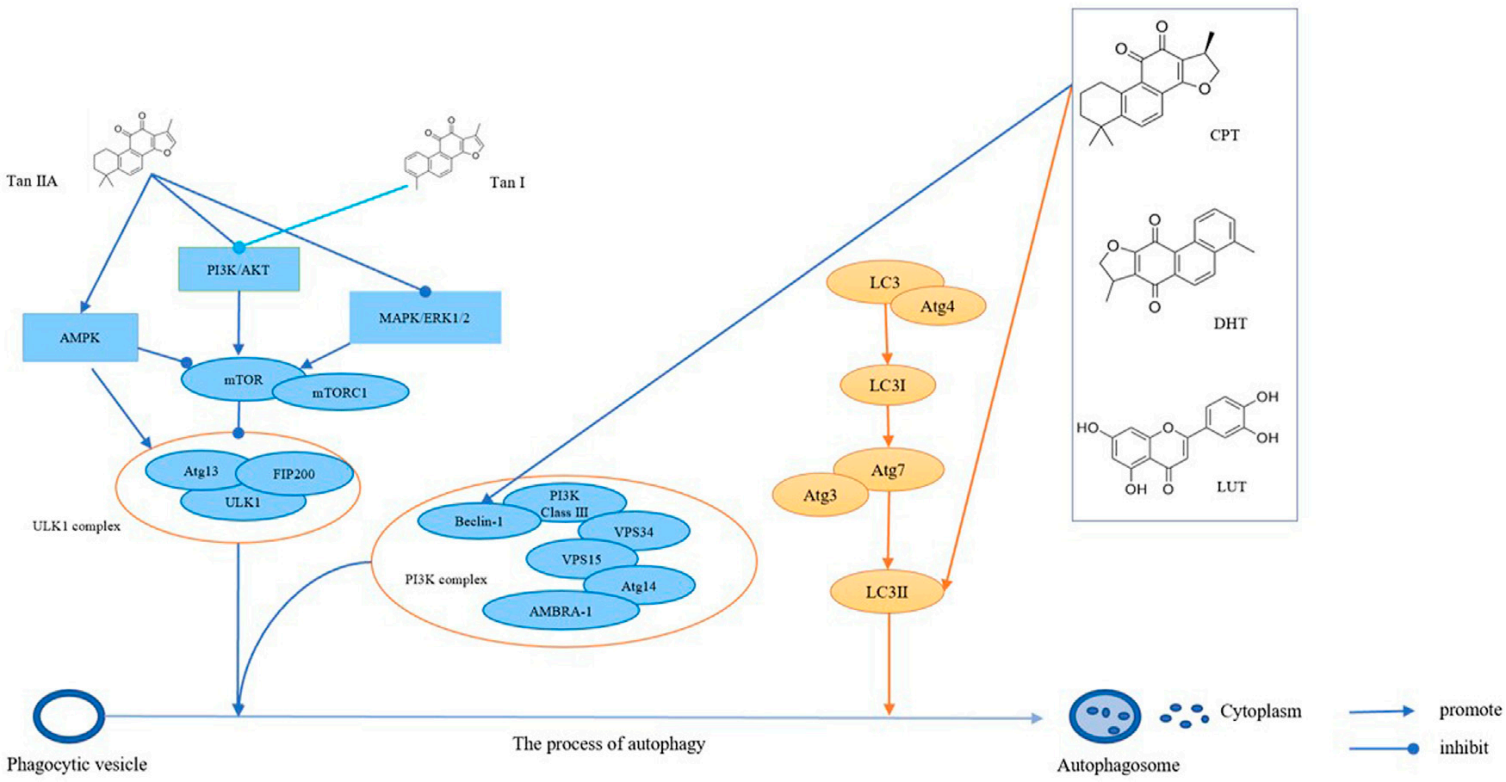
The bioactive compounds of *S. miltiorrhiza* promote autophagy in cancers. Tan IIA and Tan I regulate AMPK signaling, PI3K/AkT signaling, and MAPK/ERK1/2 signaling, thereby regulating mTOR and ULK1 complexes, which promotes the formation of autophagosomes. CPT, DHT, and LUT regulate the PI3K complex and LC3, which in turn promotes autophagosome formation.

In summary, tan IIA inhibits the mTOR pathway through different mechanisms and increases the expression of autophagy-related proteins, such as LC3-II and Beclin 1, thereby inducing autophagy in tumor cells (Figure 3).

## Targeting microRNAs (miRNAs)

MiRNAs are a class of non-coding small RNAs with a length of 21–25 nucleotides, and they are widely present in animal and plant cells (Mohr and Mott 2015). MiRNAs bind to the 3′-untranslated region (UTR) of messenger RNA (mRNA) to inhibit mRNA translation or induce mRNA degradation, thereby silencing gene expression at the post-transcriptional level (Lee and Dutta 2009; Wu et al., 2021). In malignant tumors, aberrant amplification, deletion, mutation, and epigenetic silencing of miRNAs have been identified to exert oncogenic or tumor-suppressive effects depending on the target (Rupaimoole and Slack 2017). As an epigenetic modification, miRNAs up- or downregulate the expression of downstream genes through targeting the promoter region of the target gene, thereby mediating the progression of indicated tumors (Ali Syeda et al., 2020). Thus, miRNAs may be used as diagnostic, prognostic, and predictive biomarkers of tumors, and targeting microRNAs may be a potential therapeutic approach for cancers (Iorio and Croce 2012). Studies have demonstrated that *S. miltiorrhiza* bioactive compounds mediate tumorigenesis by regulating the biological behaviors of miRNAs in cell proliferation, apoptosis, invasion, and metastasis.

### Targeting miRNAs to inhibit proliferation

It has been reported that miRNAs target proliferation-related genes and proteins, such as PKM, Akt, and MEF2D, to regulate tumor cell proliferation (Xu et al., 2020). Tan IIA suppresses the proliferation of AML cells (HL-60 and THP-1), glioma cells, esophageal cancer cells (EC109), breast cancer cells, and NSCLC cells through targeting miR-497-5p/Akt3, miR-122/PKM2, miR-16-5p/TLN1, miR-125b/STAR13, and let-7a-5p/aurora kinase A (AURKA), respectively (Zhang et al., 2016; Liu et al., 2020a; Liu et al., 2020; Nie et al., 2020; You et al., 2020; Li et al., 2022). Moreover, tan I, CPT, and tan IIA share common targets, including miR-137 and let-7a-5p, which function in inhibiting the proliferation of NSCLC by regulating the expression of ULK2/IBTK and AURKA, respectively; these common targets may be a result of highly similar chemical structures (Zhang et al., 2016; Liu et al., 2020a). It has been reported that tan I and CPT show inhibitory effects in NSCLC through targeting AURKA mediated by miR-32 (Ma et al., 2015). In addition, studies have reported that CPT inhibits the proliferation of NSCLC cells through targeting miR-146a-5p/EGFR (Qi et al., 2019). Furthermore, UA downregulates miR-499a-5/secreted frizzled related protein 4 (sFRP4) and miR-149-5p/myeloid differentiation primary response 88 (MyD88), thereby inhibiting the proliferation of NSCLC cells (Chen et al., 2020; Mandal et al., 2021). Another active ingredient of salvia, OA, suppresses the proliferation of A549 and GC cells through increasing the expression of miR-122/CCNG1/MEF2D and miR-98-5p/IL-6, respectively (Zhao et al., 2015; Xu et al., 2021). Additionally, studies have shown that LUT regulates the levels of miR-203/Ras/Raf/MEK/ERK, miR-8080/AR-V7 and miR-6809-5p/flotillin 1, which in turn inhibits the proliferation of breast cancer, castration-resistant prostate cancer (CRPC), and HCC, respectively (Gao et al., 2019; Yang et al., 2019; Naiki-Ito et al., 2020).

Researchers have also indicated that abnormal miRNAs promote tumor cell proliferation by regulating the expression of cell cycle-related proteins (Mens and Ghanbari 2018). Studies have shown that UA inhibits the proliferation of BGC-823 cells by upregulating miR-133a and inhibiting Akt1 expression, thereby promoting cell cycle arrest in the G phase (Xiang et al., 2014). Zhang et al. (2016b) reported that tan IIA induces the upregulation of miR-122 expression and inhibits the expression of pyruvate kinase M2 (PKM2) in human EC109 cells, which induces human EC109 tumor cells to arrest in S phase, thereby exerting an anticancer effect (Zhang et al., 2016).

### Targeting microRNAs to promote apoptosis

Specific miRNAs directly or indirectly participate in the regulation of multiple apoptotic pathways by regulating the level of downstream genes, and they can play an oncogenic or tumor suppressor role (Bejarano et al., 2021). Tan IIA targets miR-125b/gasdermin D (GSDMD) and miR-145/caspase 1, which induces apoptosis of nasopharyngeal carcinoma (NPC) cells (HK1 cells) and cervical carcinoma cells (HeLa cells), respectively (Tong et al., 2020; Wang et al., 2021c). In addition, tan IIA targets miR30b, further upregulating p53 levels, which induces apoptosis and death of HCC cells (Ren et al., 2017). Studies have found that tan I and UA target miR135a-3p/DR5 (prostate cancer), miR-21 (glioma), and let7b (malignant mesothelioma), which mediates the activation of caspase 3, thereby inducing tumor cell apoptosis and proliferation (Wang et al., 2012; Shin et al., 2014; Sohn et al., 2016). It has been reported that UA increases the level of miR-4500, which suppresses the expression of p-STAT3 and cleaved PARP, thereby inducing CRC cell apoptosis and inhibiting CRC growth (Kim et al., 2018).

### Targeting microRNAs to inhibit migration and invasion

Studies have found that miRNAs play an important role in tumor invasion and metastasis by affecting indicated targets and pathways (Wang and Chen 2019). Numerous studies have demonstrated that *S. miltiorrhiza* bioactive compounds regulate the levels of miRNAs, thereby inhibiting metastasis and invasion of tumor cells. LUT upregulates miR-203/Ras/Raf/MEK/ERK, which upregulates E-cadherin and downregulates N-cadherin, thereby inhibiting the progression of EMT and preventing tumor cell invasion and metastasis of breast cancer (Gao et al., 2019). Furthermore, LUT upregulates miR-384 and subsequently downregulates the expression of multivitamin trophic factor (PTN), which inhibits CRC and osteosarcoma (OS) cell migration and invasion (Yao et al., 2019; Qin et al., 2022). In addition, LUT inhibits migration- and invasion-related factors, including VEGF, MMP2, and MMP9, by upregulating miR-133a-3p levels, thereby suppressing invasion and metastasis of NSCLC (Pan et al., 2022). In addition, UA targets let 7b, which inhibits the expression of p-Akt, β-catenin, and Twist, thereby repressing the EMT process (Sohn et al., 2016).

In summary, *S. miltiorrhiza* bioactive compounds inhibit tumor cell proliferation, migration, and invasion as well as induce apoptosis by regulating the expression of miRNAs, thereby exerting antitumor effects (Table 1).

**TABLE 1:** Regulation of microRNAs by *S. miltiorrhiza* bioactive compounds in cancer progression inhibition.

Bioactive compounds of *S. miltiorrhiza*	Physiological effects	Cancer types	miRNAs	MiRNA targets	References
Regulation of microRNA by active ingredients of tanshinones					
Tan IIA	Inhibit proliferation	Esophageal cancer cells (EC109)	miR-122**↑**	PKM2**↓**	Zhang et al. (2016b)
		AML cells (HL-60 and THP-1)	miR-497-5p**↑**	AKT3**↓**	Nie et al. (2020)
		Glioma cells	miR-16-5p**↑**	TLN1**↓**	You et al. (2020)
Tan IIA	Inhibit proliferation	Breast cancer cells	miR-125b**↓**	STARD13**↓**	Li et al. (2022a)
		NSCLC	miR-137**↑**	ULK2 and IBTK**↓**	Zhang et al. (2016a), Liu et al. (2020)
		NSCLC	Let-7a-5p**↑**	AURKA**↓**	Zhang et al. (2016a), Liu et al. (2020)
	Induce apoptosis	Nasopharyngeal carcinoma (NPC)	miR-125b**↓**	caspase 1 **↑**	Wang et al. (2021c)
		HepG2 cells	miR30b**↓**	p53**↑**	Ren et al. (2017)
		HeLa cells	miR-145**↑**	GSDMD**↑**	Tong et al. (2020)
Tan I	Inhibit proliferation	NSCLC	miR-137**↑**	ULK2 and IBTK**↓**	Zhang et al. (2016a), Liu et al. (2020)
		NSCLC	Let-7a-5p**↑**	AURKA**↓**	Zhang et al. (2016a), Liu et al. (2020)
Tan I	Inhibit proliferation	NSCLC	miR-32**↑**	AURKA**↓**	Ma et al. (2015)
	Induce apoptosis	Prostate cancer cells	miR135a-3p**↑**	DR5**↑**	Shin et al. (2014)
CPT	Inhibit proliferation	NSCLC	miR-137**↑**	AURKA**↓**	Zhang et al. (2016a), Liu et al. (2020)
		NSCLC	Let-7a-5p**↑**	AURKA**↓**	Zhang et al. (2016a), Liu et al. (2020)
		NSCLC	miR-32**↑**	AURKA**↓**	Ma et al. (2015)
		NSCLC	miR-146a-5p**↑**	EGFR**↓**	Qi et al. (2019)
Regulation of microRNA by active ingredients of triterpenoids					
OA	Inhibit proliferation	GC	miR-98-5p**↑**	IL-6**↓**	Xu et al. (2021)
		A549	miR-122**↑**	CCNG1 and MEF2D**↓**	Zhao et al. (2015)
UA	Inhibit proliferation	NSCLC	miR-499a-5**↓**	sFRP4**↓**	Mandal et al. (2021)
		NSCLC	miR-149-5p**↓**	MyD88**↓**	Chen et al. (2020)
		BGC-823 cells	miR-133a**↑**	Akt1**↓**	Xiang et al. (2014)
	Induce apoptosis	glioma cells	miR-21**↓**	caspase-3**↑**	Wang et al. (2012)
		CRC cells	miR-4500**↑**	STAT3**↓**	Kim et al. (2018)
		Malignant mesothelioma	let7b**↑**	caspase 3**↑**	Sohn et al. (2016)
	Inhibit invasion and metastasis	Malignant mesothelioma	let7b**↑**	AKT, β-catenin and Twist **↓**	Sohn et al. (2016)
Regulation of microRNA by active ingredients of flavonoids					
LUT	Inhibit proliferation	Breast cancer	miR-203**↑**	Ras/Raf/MEK/ERK**↓**	Gao et al. (2019)
		Castration-resistant prostate cancer (CRPC)	miR-8080**↑**	AR-V7**↓**	Naiki-Ito et al. (2020)
LUT	Inhibit proliferation	Hepatocellular carcinoma (HCC)	miR-6809-5p**↓**	flotillin 1**↓**	Yang et al. (2019)
	Inhibit invasion and metastasis	Breast cancer	miR-203**↑**	Ras/Raf/MEK/ERK**↓**	Gao et al. (2019)
		CRC cells	miR-384**↑**	PTN**↓**	Yao et al. (2019a)
		Osteosarcoma (OS) cell	miR-384**↑**	PTN**↓**	Qin et al. (2022)
		NSCLC	miR-133a-3p**↑**	PURB**↓**	Pan et al. (2022)

## Regulation of epigenetic enzymes

Epigenetic mechanisms refer to heritable changes in gene expression without altering the DNA sequence, and they include DNA methylation, chromatin remodeling, histone modifications, and non-coding RNAs (Kanwal et al., 2015; Huang et al., 2021b). Abnormal epigenetic alterations are often involved in tumor initiation, progression, and metastasis (Esteller 2008). *S. miltiorrhiza* bioactive compounds have been reported to inhibit tumorigenesis and progression by modulating indicated epigenetic mechanisms, including DNA methylation, histone acetylation, ubiquitination, and DNA topoisomerase (Tian et al., 2018; Bhattacharjee and Dashwood 2020). For example, DHT promotes Keap1-mediated degradation of nuclear factor erythroid 2-related factor 2 (Nrf2) ubiquitination, and it increases the accumulation of ROS, which in turn downregulates Bcl-2, cyclin B1, and Cdc2 levels as well as upregulates Bax levels in ovarian cancer cells *in vivo* and *in vitro* (Sun et al., 2022).

### DNA methylation

In normal cells, DNA methylation occurs mainly in repetitive genomic regions and is responsible for silencing gene expression and maintaining genome stability (Sandoval et al., 2011). However, special DNA regions, such as promoters, especially CpG islands (CpGs), are often unmethylated (Bird 1986). Hypomethylation in tumor cells is mainly due to methylation deficiency in repetitive genomic regions, leading to genomic instability and activation of transposon promoters, whereas hypermethylation is the ab initio methylation of CpGs, which can lead to transcriptional silencing of growth regulatory genes (Lakshminarasimhan and Liang 2016). Cancer cells display genome-wide hypomethylation and site-specific CpG promoter hypermethylation (Rodríguez-Paredes and Esteller 2011). Abnormal increased DNA methylation leads to transcriptional repression and decreased gene expression (Pan et al., 2018). Studies have reported that *S. miltiorrhiza* bioactive compounds regulate the level of methylation in tumor cells and inhibit tumor development (Kim et al., 2016; Li et al., 2022). For example, tan IIA, LUT, and UA target the abnormal hypermethylation at the CpG methylation region in the Nrf2 promoter by reducing the expression of DNA methyltransferases (DNMTs), including DNMT1, DNMT3a, and DNMT3b, thereby inhibiting cell proliferation of skin and colon cancers (Wang et al., 2014; Kim et al., 2016; Zuo et al., 2018). Moreover, it has been reported that the inhibition of NRF2 promoter methylation is mediated by tan IIA *via* ten-eleven translocation (TET)-2 activation (Yang et al., 2020). In addition, OA decreases the promoter DNA demethylation of programmed death-ligand 1 (PD-L1) by inhibiting the expression of TET-3, which downregulates PD-L1, ultimately enhancing T cell-mediated killing of MKN-45 cells (Lu et al., 2021). Moreover, it has been reported that Sal B induces DNMT1-mediated demethylation of the PTCH1 gene, which in turn inhibits hepatic stellate cell (HSC) EMT *in vivo* and *in vitro* (Yu et al., 2015).

### Histone methylation

Histone methylation is a major player in the regulation of gene expression and genetic stability, and dysfunction of histone methylation contributes to the development of various cancers (Rotili and Mai 2011). Methylation occurs primarily on lysine or arginine residues of histones H3 and H4, which is controlled by histone methyltransferases (HMTs) and histone demethylases (HDMs) (Janardhan et al., 2018). Polycomb repressive complex 2 (PRC2) is a complex that mediates gene silencing by regulating chromatin structure (Chan and Morey 2019). Enhancer of zeste homolog 2 (EZH2), a polycomb group (PcG) protein, is an enzymatic catalytic subunit of PRC2 that regulates gene expression through catalyzing primarily tri-methylation of histone H3 Lys-27 (H3K27), and it functions in the development and progression of a variety of cancers, such as breast cancer (Bachmann et al., 2006), GC (Gan et al., 2018), and NPC (Duan et al., 2020). It has been reported that tanshindiol C inhibits EZH2 activity, which in turn downregulates H3K27me3 levels, thereby inhibiting the proliferation of the Pfeiffer cell line, a type of B cell lymphoma (Woo et al., 2014). Furthermore, tan I directly binds to EZH2, which inhibits PRC2 activity, resulting in a decrease in tri-methylated modification of H3K27, and this decrease, in turn, upregulates MMP9 and ABCG2, ultimately inhibiting the proliferation of human leukemia cells *in vitro* and *in vitro* (Huang et al., 2021). Moreover, tan I reduces the expression of CCAAT-enhancer-binding protein β (C/EBPβ), which inhibits the mRNA levels of the JMJD2b histone H3K9 demethylase as well as cell cycle-related genes, further regulating the mitotic clonal expansion (MCE) process (Jung et al., 2020). Lysine specific demethylase 1 (LSD1) is a flavin adenine dinucleotide (FAD)-dependent amine oxidase (AO) that catalyzes the demethylation of mono- and di-methyl on H3K4 and H3K9, triggering transcriptional repression and activation, respectively (Karakaidos et al., 2019). Studies have demonstrated that CPT blocks the interaction of LSD1 and androgen receptor (AR) at the promoter of the AR target gene, prostate-specific antigen (PSA), which demethylates H3K9me1/2, thereby inhibiting the transcriptional activity of PSA and suppressing the proliferation of AR-positive prostate cancer cells (Wu et al., 2012). Moreover, tan IIA upregulates the level of H3K27me1 and H3K4m2 on the *TP53* promoter by inhibiting LSD1, thereby activating the transcriptional activity of p53 (Zheng et al., 2018). It has been reported that the mRNA and protein levels of p53 are downregulated in several types of tumors, such as prostate cancer and colon cancer (Bossi and Sacchi 2007). Therefore, reactivating p53 is an important strategy for tumor therapy. Taken together, these findings suggest that tan IIA may increase the level of H3K27me1 and H3K4m2 on the p53 promoter by targeting LSD1, which activates p53, thereby promoting tumor cell cycle arrest and apoptosis.

### Histone acetylation

Histone acetylation is a reversible and homeostatic process regulated by histone acetyltransferases (HATs) and histone deacetylases (HDACs) (Attoub et al., 2011). Studies have reported abnormal acetylation levels in certain tumors (Zhao and Shilatifard 2019). Histone lysine residues are hyperacetylated in drug-resistant prostate cancer, acute myeloid leukemia, gastrointestinal stromal tumors, lymphomas, and TNBC (He et al., 2021), whereas acetylation levels are decreased in ovarian cancer, colon cancer, liver cancer, and skin cancer (Wu et al., 2020). Overexpression of HDAC is present in tumors with low acetylation levels, indicating that targeting HDAC may be a strategy for treatment of these tumors (Li and Seto 2016). To date, numerous HDAC inhibitors are in various stages of clinical research; vorinostat (SAHA), beristatin (PXD101), and bilestat (LBH589) are approved for the clinical treatment of cutaneous T cell lymphoma, peripheral T cell lymphoma and multiple myeloma, respectively (Ho et al., 2020), and other HDAC inhibitors have been investigated in clinical trials for the treatment of various cancers (Ono et al., 2018; Xie et al., 2018). *S. miltiorrhiza* bioactive compounds have been found to target histone deacetyltransferases (HDACs) and identified as potential inhibitors of HDACs. Tan IIA suppresses the expression and enzymatic activity of HDAC1, HDAC3, HDAC4, and HDAC8, which increases histone 3 acetylation and RNA polymerase II recruitment to the Nrf2 transcription start site, thereby inhibiting TPA-induced JB6 P+ cell neoplastic transformation (Wang et al., 2014). In contrast, LUT increases the enrichment of H3K27ac in the promoter region of Nrf2 by downregulating HDAC1, HDAC2, HDAC3, HDAC6, and HDAC7 expression and enzymatic activity, further promoting the transcriptional activity of the Nrf2 gene, which in turn inhibits the cellular activity of HCT116 (Zuo et al., 2018).

The level of acetylation is closely related to tumor stage, metastasis, and invasion as well as prognosis (He et al., 2021). Aurora A is a mitotic S/T kinase involved in the process of cell division, and the expression of aurora A is elevated in various tumors (Meraldi et al., 2004). Tan I reduces the level of histone acetylation at the aurora A DNA promoter region, which downregulates aurora A and ultimately inhibits the growth of breast cancer cells (Gong et al., 2012). Furthermore, LUT reduces the level of H3K27ac and H3K56ac at the MMP9 promoter region by inactivating the Akt/mTOR signaling pathway, thereby inhibiting metastasis of AR-positive TNBC (Wu et al., 2021).

### Ubiquitination

Ubiquitination is an important post-translational modification mediated by ubiquitin-activating enzymes (E1), ubiquitin-conjugating enzymes (E2), and ubiquitin ligases (E3) (Cockram et al., 2021). Abnormal ubiquitination alters intracellular physiological functions, leading to the initiation and development of cancer (Pellegrino et al., 2022). For example, overexpression of the E3 ligase, mouse double minute 2 (MDM2), mediates ubiquitination of p53 and promotes proteasomal degradation of p53, which inhibits p53 function, leading to tumorigenesis and cancer cell progression (Wang et al., 2017). It has been reported that tan IIA induces ubiquitous degradation of the anti-apoptotic protein, Mcl-1, through targeting the EGFR-Akt signaling pathway, which induces NSCC cell apoptosis (Gao et al., 2020). Tan IIA promotes the FBW7 E3 ligase-mediated ubiquitination degradation of c-Myc, which induces OSCC cell apoptosis (Chen et al., 2020a; Li et al., 2020). Moreover, tan I and DHT activate the Nrf2 pathway by reducing Nrf2 ubiquitination, thereby maintaining redox homeostasis of skin fibroblasts and HBE cells (Tao et al., 2013a; Tao et al., 2013).

**FIGURE 4:**
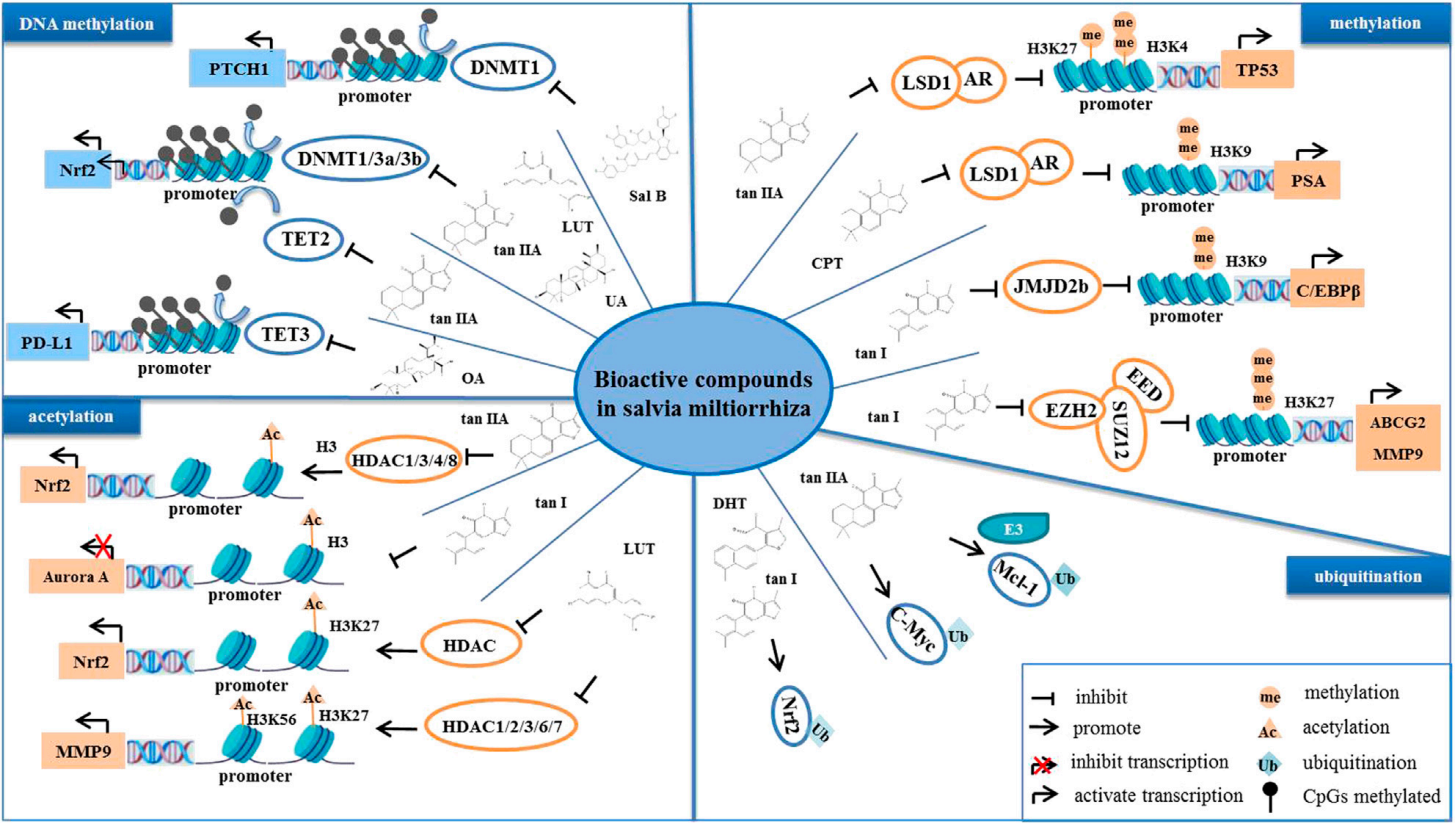
The bioactive compounds of *S. miltiorrhiza* regulate epigenetic modifications. *S. miltiorrhiza* bioactive compounds suppress the transcriptional activity and enzymatic activity of their target genes by regulating epigenetic modification-related enzymes.

In summary, *S. miltiorrhiza* bioactive compounds suppress tumor growth and development by regulating DNA methylation, histone methylation, acetylation, and ubiquitination to silence tumor suppressors or promote oncogenes (Figure 4).

## Future prospects

Currently, chemotherapy and radiotherapy are the main methods for the clinical treatment of tumors, but these methods have serious shortcomings, such as resistance and toxic side effects. Thus, combination therapy may be important to improve tumor sensitivity. We previously demonstrated that the bioactive compounds of *S. miltiorrhiza* inhibit cancer cell proliferation, induce apoptosis, suppress metastasis, and suppress invasion. Thus, we speculate that *S. miltiorrhiza* bioactive compounds may be used as adjuvant drugs to improve the sensitivity of drug-resistant tumors.

At present, drug resistance is one of the major barriers to cancer treatment, which limits the efficacy of chemotherapy drugs, leading to tumor recurrence, metastasis, and cell death resistance (Haider et al., 2020). Doxorubicin (Dox) and paclitaxel are first-line treatments for patients with metastatic and early-stage breast cancer (Abu Samaan, Samec et al., 2019; Jamialahmadi et al., 2021). However, it has been reported that patients develop resistance to these drugs during the treatment of breast cancer, greatly affecting the efficacy of the drugs (Rivera and Gomez 2010). Moreover, platinum drugs, such as cisplatin and oxaliplatin (OXA), induce DNA damage in tumor cells; they are mainly used in the treatment of ovarian cancer, but their efficacy is severely limited due to drug resistance (Pujade-Lauraine et al., 2019). Studies have found that the bioactive compounds of *S. miltiorrhiza* have potential roles in reversing resistance and enhancing chemotherapeutic drug sensitivity. For example, tan IIA increases the sensitivity of OXA-resistant CRC cells to OXA by regulating the Akt/ERK signaling pathway, thereby enhancing the anti-proliferation effect and inducing apoptosis in SW480/OXA and HT29/OXA cells (Zhang et al., 2019b). Furthermore, CPT/DHT reduces the P-glycoprotein (P-gp) mRNA and protein levels as well as inhibits P-gp ATPase activity of SW620 Ad300 cells (Hu et al., 2014). Moreover, tan IIA reduces the accumulation of β-catenin in the nucleus by repressing β-catenin nuclear translocation, which enhances Dox inhibition of MCF-7/Dox cell proliferation and migration (Li et al., 2021). Adenosine triphosphate (ATP)-binding cassette (ABC) transporters, such as P-gp, breast cancer resistance protein (BCRP), and multidrug resistance-related protein 1 (MRP1), catalyze the transduction of structurally diverse compounds across cell membranes *via* ATP-dependent transport (Zahra et al., 2020). Because ABC transporters are overexpressed in tumor cells, they pump out the chemotherapeutic drugs, thereby inducing tumor cells to acquire drug resistance (Choi and Yu 2014). It has been reported that tan IIA downregulates the expression of P-gp, BCRP, and MRP1, further promoting intracellular accumulation of Dox, which induces apoptosis and suppresses proliferation of MCF7/Dox cells, thereby alleviating the inhibitory effect of Dox on the hematopoietic function and Dox-induced cardiotoxicity and nephrotoxicity (Li et al., 2019).

*S. miltiorrhiza* bioactive compounds not only increase the sensitivity of drug-resistant cells to chemotherapeutic drugs but also synergistically inhibit tumor development. Bai et al. reported that tan IIA combined with 5-FU represses the activation of NF-κB and inhibits CRC cell proliferation (Bai et al., 2016). Furthermore, tan IIA and Dox cotreatment decreases the activity of the VEGF/PI3K/Akt signaling pathway, which promotes apoptosis and induces cell cycle arrest, thereby suppressing proliferation, metastasis, and invasion of A549 cells (Xie et al., 2016). Moreover, tan I and paclitaxel cooperate to promote apoptosis and accelerate cell senescence, thereby inhibiting proliferation and metastasis of ovarian cancer cells (A2780 and ID-8 cells) (Zhou et al., 2020). In addition, the combination of LUT and OXA induces apoptosis and cell cycle arrest as well as inhibits proliferation of SGC-7901 gastric cancer cells (Ren et al., 2020).

Radiotherapy is an effective method of cancer treatment, but the resistance and side effects of radiation therapy affect its efficacy. Therefore, sensitizers that kill tumor cells improve the efficacy of radiotherapy (Chevalier 2020). Tan IIA combined with irradiation reduces histone H3 (S10) phosphorylation in ionizing radiation-resistant cell lines (CAL27-IR and SCC25-IR cells), and this combination enhances irradiation-induced DNA damage and promotes apoptosis, thereby inhibiting tumor growth *in vivo* and *in vitro* (Li et al., 2021). Moreover, tan IIA increases radiation-induced ROS production and induces autophagy, which in turn increases the sensitivity of OSCC cells (SCC090 cells) to radiation (Ding et al., 2016). Pro-oncogenic protein phosphoribosyl pyrophosphate aminotransferase is an essential enzyme that catalyzes the first step in purine biosynthesis and promotes the increase in purine metabolism in cancer cells, and its overexpression promotes cancer cell proliferation and invasion. Tan I downregulates the expression of PPAT, which in turn enhances the radiosensitivity of radiation-resistant lung cancer cells (H358-IR and H157-IR cells), thereby inhibiting cell proliferation (Yan et al., 2018).

**FIGURE 5:**
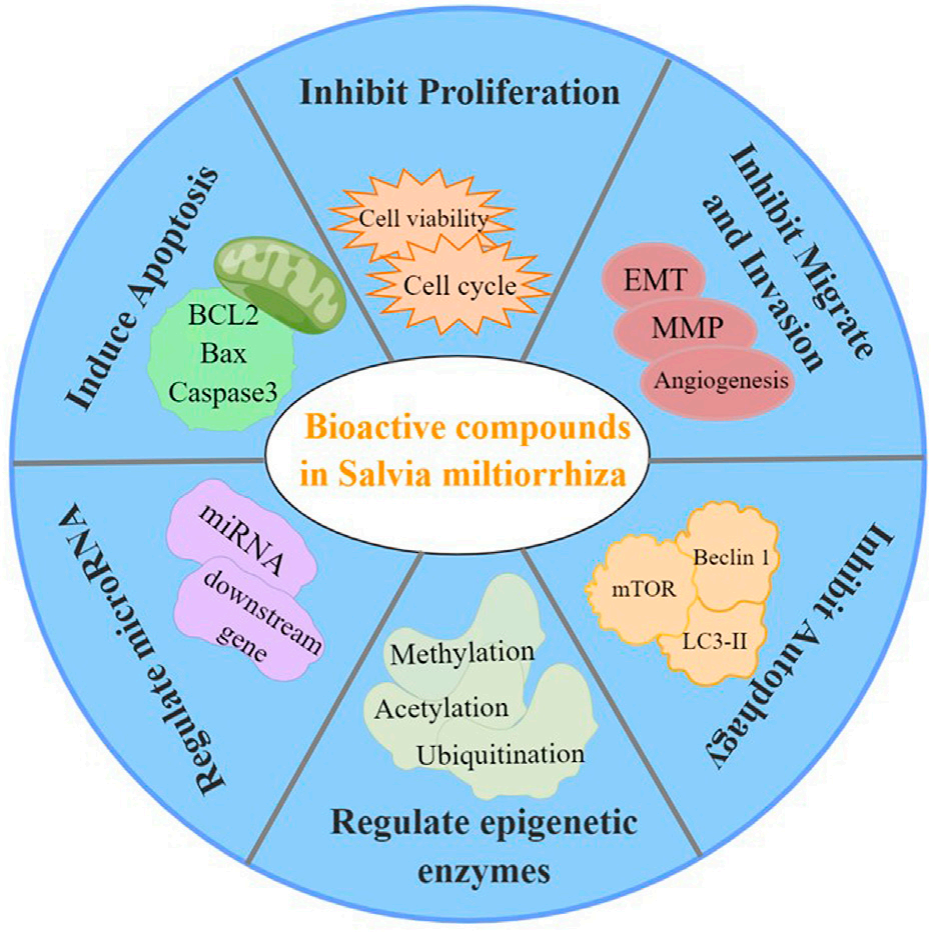
Pharmacological activities of *S. miltiorrhiza* bioactive compounds resulting in antitumor effects.

In summary, the bioactive compounds of *S. miltiorrhiza* target miRNAs and epigenetic enzymes as well as function to inhibit cancer cell proliferation, induce cell cycle arrest, induce apoptosis, trigger autophagy, suppress metastasis, and suppress invasion (Figure 5). Moreover, *S. miltiorrhiza* bioactive compounds have multiple roles as adjuvant drugs in chemotherapy and radiotherapy as follows: 1) improve the sensitivity of drug-resistant cells to chemotherapeutic drugs; 2) work together with chemotherapeutic drugs to enhance the therapeutic effect; and 3) combine with radiotherapy to improve the effect of radiotherapy (Table 2). Thus, *S. miltiorrhiza* bioactive compounds have an antitumor effect and potential application prospects in tumor treatment and adjuvant medicine. In addition, one critical mechanism of *S. miltiorrhiza* bioactive compounds in suppressing cancer initiation and progression is through targeting miRNAs, including miR-125b, miRNA-122, and miRNA-384, which are present in various cancer cells, suggesting that these miRNAs may play important roles in cancer progression. The pharmacological activity and mechanism of *S. miltiorrhiza* bioactive compounds are continually being elucidated, thereby supporting their potential application in future clinical cancer therapies.

**TABLE 2:** Effect of *S. miltiorrhiza* bioactive compounds combined with chemotherapy and radiotherapy.

Chemical composition	Combination drugs	Mechanism	Resistant cells	Physiological effect	References
Bioactive compounds of S. miltiorrhiza improves sensitivity of drug-resistant tumors					
Tan IIA	Oxaliplatin	Regulate Akt/ERK pathway, decrease the levels of Bcl-2, p-Akt and p-ERK, and increase the levels of Bax and active caspase 3	Oxaliplatin-resistant colon cancer cell lines (SW480/OXA and HT29/OXA)	Anti-proliferation	Zhang et al. (2019b)
Tan IIA	Doxorubicin	Inhibit β-catenin nuclear translocation, decrease the expressions of c-Myc, E-cadherin, MMP-2 and MMP-9	Doxorubicin-resistant breast cancer cell (MCF-7/dox)	Suppress proliferation and migration	Li et al. (2021a)
	Doxorubicin	Downregulate MRP1expression, arrest cell cycle at G2/M phase, down-regulate BCL2 and up-regulate BAX and p53	Doxorubicin-resistant gastric cancer cell lines (SNU-719R and SNU-620)	Arrest cell cycle induce apoptosis	Xu et al. (2018)
	Doxorubicin	Downregulate the expression of P-gp, BCRP and MRP1	Doxorubicin-resistant breast cancer cell (MCF7/dox)	Inhibit proliferation, induce apoptosis	Li and Lai (2017), Li et al. (2019a)
		Upregulate PTEN, activate AKT			
	Gefitinib	Regulate VEGFR2/Akt pathway	Gefitinib-resistant cell lines (HCC827/gefitinib and PC-9/gefitinib)	Suppress proliferation, migration and invasion	Wang et al. (2019a)
	Taxol	Reduce the expression of microtubule-associated protein (Tau)	Taxol tolerant MCF-7 cells (MCF/Taxol)	Inhibit cell viability	Lin et al. (2018)
CPT	Doxorubicin and irinotecan	Reduce the level of P-gp mRNA and protein, inhibit P-gp ATPase activity	Doxorubicin and irinotecan- resistant cells (SW620 Ad300)	Inhibit cell viability	Hu et al. (2014)
DHT	Doxorubicin and irinotecan	Reduce the level of P-gp mRNA and protein, inhibit P-gp ATPase activity	Doxorubicin and irinotecan- resistant cells (SW620 Ad300)	Inhibit cell viability	Hu et al. (2014)
DHT I	Caclitaxel	Decrease ABCB1 and NF-κB expression, reduce NF-κB activity	Paclitaxel-resistant anaplastic thyroid cancer cells (SW1736 and 8505C)	Inhibit proliferation	Allegri et al. (2021)
LUT	Cisplatin (DDP)	Downregulate BCL2 expression	Cisplatin-resistant ovarian cancer cells (CAOV3/DDP)	Suppress proliferation, migration and invasion, reduce apoptosis	Wang et al. (2018)
Synergistic therapy with bioactive compounds of S. miltiorrhiza and chemotherapeutic drugs					
Tan IIA	5-FU	Repress the activation of NF-κB	HCT1116 and COLO205 cells	Inhibit cell proliferation	Bai et al. (2016)
	Adriamycin	Decrease the activity of VEGF/PI3K/Akt signaling pathway	A549 and PC9 cells	Suppress proliferation, metastasis and invasion, promote apoptosis	Xie et al. (2016)
Tan IIA	5-FU	Downregulate P-gp, LC3-II, VEGF, MMP-7 and NF-κB p65 protein expression	Colo205 cells	Repress tumor growth	Su (2012)
	Paclitaxel	Upregulate the expression of γ-H2AX, p21, p16 and Bax, downregulate the expression of BCL2	Ovarian cancer cells (A2780 and ID-8)	Inhibits proliferation and metastasis	Zhou et al. (2020a)
	Nutlin-3	Regulate MDM2-P53 and AKT/mTOR pathway, induce cell cycle arrest at S and G2 phase	ALL cell lines (SUP-B15, NALM-6, HL-60 and MV4-11)	Suppress cell viability reduce apoptosis and autophagy	Guo et al. (2019)
	Doxorubicin	Increase LDH leakage, and the level of ROS and NADPH oxidase 4 activity, meantime decrease the expression of SOD1	HepG2 cells	Repress cell viability	Kan et al. (2014)
		Upregulate the expression of caspase3			
	Nutlin-3 and imatinib	Inhibit AKT/mTOR pathway and reactivation of p53 pathway arrest cell cycle at S phase	ALL cell lines	Inhibit cell viability	Guo et al. (2017)
Tan IIA	Cisplatin	Increase the expression of cleaved caspase 3 and cleaved PARP, decrease the expression of survivin	FaDu cells	Suppress proliferation	Zhao et al. (2017a)
	As2O3	Arrest cell cycle at G0/G1 phase, reduces the expression of Pgp	NB4 cells, MR2 cells	Inhibit proliferation	Li et al. (2009), Zhang et al. (2010)
DHT	Temozolomide	Reduce NF-kB activity	Glioblastoma cells (U87-MG (MGMT-) and T98G (MGMT+))	Educe proliferation	Kumar et al. (2019)
		Arrest cell cycle at G0/G1 phase			
Tanshinone	Carboplatin	Increase the percentage of CD4þ and CD8þ subsets and the activity of NK and CTL	B16 cells	Inhibit tumor growth	Wu et al. (2016)
LUT	Indole-3-Carbinol	Regulate SIRT1/ERα pathway	ER^+^ breast cancer MCF7 and T47D cells	Repress proliferation	Wang et al. (2021b)
		Arrest cell cycle at G1phase			
	Paclitaxel	Inhibit the expression of Nrf2, HO-1, Sirt3 and Cripto-1	MDA-MB-231 cells	Inhibit cell viability	Tsai et al. (2021)
	Vaccinia virus (VV) that harbors IL-24 (VV-IL-24)	Upregulate IL-24 gene expression	Liver cancer cell lines (MHCC97-H, HepG2, PLC/PRF/5, Hep3B and HEK293)	Inhibit the development of tumor induce apoptosis	Wang et al. (2021)
LUT	Lapatinib	Upregulate the expression of FOXO3a and NQO1 and their downstream target genes Bim, GADD45, P21, and decrease the phosphorylation level of FOXO3a protein	Breast cancer cell lines (SKBR-3、ZR-75-1 and BT-474)	Suppress tumor growth, migration and invasion	Zhang et al. (2020)
	Myo-inositol (MI)	Decrease the expression of p-PDK1, p-Akt	A549	Inhibit proliferation and migration	Wang et al. (2018b)
		Increase cyclin D1 levels, and arrest cell cycle at G0/G1 phase			
	OXA	Upregulate the expression levels of Cyt c, cleaved caspase-3 and Bax, downregulate Bcl-2 and pro-caspase-3	SGC-7901 cells	Inhibit proliferation	Ren et al. (2020)
				Induce apoptosis	
Bioactive compounds of S. miltiorrhiza improves radiosensitivity					
Tan IIA	Ray(4Gy)	Reduce histone H3 (S10) phosphorylation, induce DNA damage	Ionizing-radiation-acquired resistance cell lines (CAL27-IR and SCC25-IR)	Promote apoptosis, inhibit tumor growth	Li et al. (2021)
	Ray(2Gy)	Increase ROS production, upregulate the protein levels of Beclin 1, Atg5 and LC3-II	Oral squamous cell carcinoma SCC090 cells	Promote autophagy	Ding et al. (2016)
Tan I	Ray(6Gy)	Own-regulate the expression of PPAT	Radiation-resistant lung cancer cells (H358-IR and H157-IR)	Inhibit cell proliferation	Yan et al. (2018)
